# The Role of P53 in Immune Evasion and Therapeutic Strategies in Hematologic Malignancies

**DOI:** 10.7150/jca.113576

**Published:** 2025-08-28

**Authors:** Jing Wen, Linlin Fu, Hui Zhong, Hongxia Chen

**Affiliations:** 1 School of Clinical Medicine, Southwest Medical University, Luzhou, Sichuan, China.; 2 Department of Hematology, Chongqing University Three Gorges Hospital, Chongqing, China.

**Keywords:** P53, Immune Evasion, Hematologic Malignancies

## Abstract

P53 is a crucial tumor suppressor gene that plays an essential role in maintaining genomic stability, regulating cell cycle progression, and inducing apoptosis. In hematologic malignancies, P53 mutations are frequently associated with poor prognosis, treatment resistance, and immune evasion. Recent research has highlighted the impact of P53 dysfunction on tumor immune escape mechanisms, including impaired antigen presentation, altered cytokine signaling, and recruitment of immunosuppressive cells. This review integrates recent findings on P53 mutations in hematologic malignancies, focusing on their role in immune evasion and potential therapeutic strategies aimed at restoring P53 function or targeting associated pathways. Understanding these mechanisms may provide new insights into the development of effective immunotherapeutic approaches for hematologic cancers.

## Introduction

Hematologic malignancies are characterized by genomic instability, often driven by mutations in tumor suppressor genes such as *TP53*. The advent of Next-Generation Sequencing (NGS) has revolutionized patient stratification by identifying *TP53* mutational status, which is critical for prognosis and therapeutic decision-making 1. For instance, in acute myeloid leukemia (AML), *TP53*-mutated patients exhibit complex karyotypes and a dismal median survival of <6 months, necessitating first-line therapies distinct from conventional chemotherapy (e.g., hypomethylating agents or targeted drugs) 2. Similarly, in myelodysplastic syndromes (MDS), *TP53* mutations correlate with high-risk subtypes and rapid progression to AML, while in chronic lymphocytic leukemia (CLL), 17p deletions co-occurring with *TP53* mutations predict resistance to BTK inhibitors 3. This stratification underscores the need for mutation-specific therapeutic approaches to improve outcomes in these high-risk cohorts.

## P53 Structure and Function

**Figure 1:**
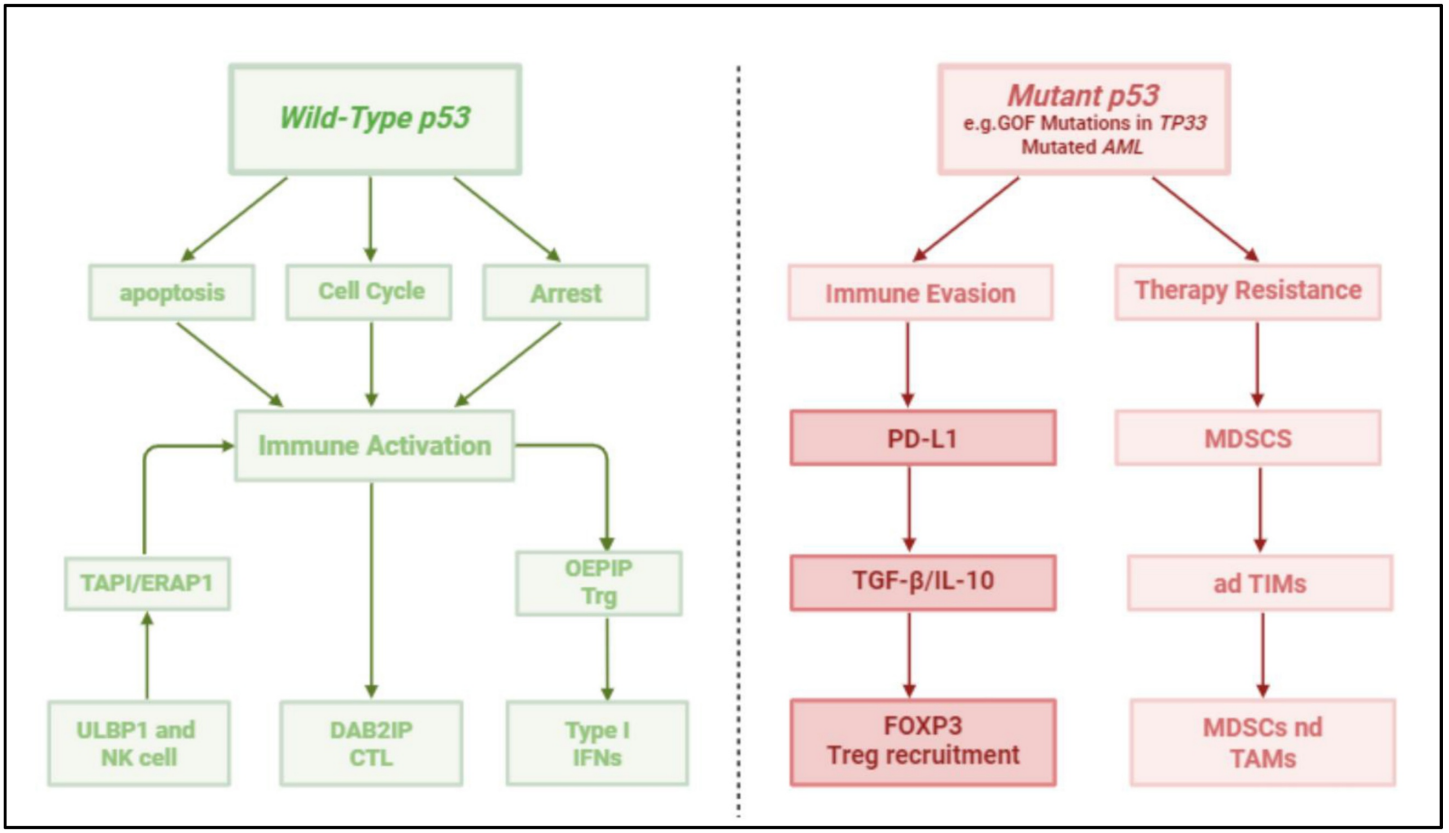
** Contrasting Roles of Wild-Type and Mutant p53 in Hematologic Malignancies.** This two-panel diagram illustrates the dual functions of p53 in the tumor microenvironment of hematologic cancers. The left panel depicts **wild-type p53**, which maintains genomic stability through induction of cell cycle arrest (via p21 and Gadd45), apoptosis (via Bax, PUMA, NOXA), and enhancement of anti-tumor immunity by promoting antigen presentation (TAP1, ERAP1) and stimulating immune effector cells (CTLs, NK cells). The right panel shows **mutant p53**, particularly gain-of-function (GOF) variants, contributing to **immune evasion** by downregulating antigen processing machinery, upregulating immune checkpoints (PD-L1), altering cytokine secretion (↑ TGF-β, IL-10, ↓ Type I IFNs), and promoting recruitment of immunosuppressive cells (Tregs, MDSCs, adTIMs: adaptive tumor-infiltrating myeloid cells). These opposing mechanisms highlight the importance of p53 status in shaping immune surveillance and therapy resistance in AML, CLL, and other hematologic malignancies.

P53 is a transcription factor that regulates the expression of hundreds of genes involved in cell cycle arrest, apoptosis, DNA repair, and immune modulation 4. Structurally, P53 consists of several functional domains: transactivation domain (TAD), proline-rich domain (PRD), DNA-binding domain (DBD), tetramerization domain (TD), and regulatory domain (RD) 5. Wild-type P53 maintains cellular homeostasis by responding to various stress signals, including DNA damage and oncogene activation 6. However, TP53 mutations often result in loss of function (LOF) or gain of function (GOF), contributing to tumorigenesis and immune escape. GOF mutations can lead to the activation of oncogenic pathways, further exacerbating tumor progression and immune suppression 7 (Figure 1, Table 1). In hematologic malignancies, TP53 mutations cluster in the DBD, with hotspot codons R175, R248, and R273 prevalent in AML and MDS. These mutations disrupt DNA binding, leading to loss of function (LOF) or gain of function (GOF). GOF mutants (e.g., R248Q) promote oncogenic signaling (e.g., NF-κB activation) and immune evasion. In AML, single-hit TP53 mutations (monoallelic) coexist with multi-hit alterations (bi-allelic mutations + 17p deletions), driving aggressive phenotypes and therapy resistance 8 (Table 2).

## Mechanisms of P53-Mediated Immune Evasion

### Impaired Antigen Presentation

P53 mutations disrupt the major histocompatibility complex (MHC) class I antigen presentation pathway, preventing immune cells from recognizing and eliminating tumor cells 9. Mutant P53 downregulates key components of antigen processing, such as transporter associated with antigen processing (TAP1) and endoplasmic reticulum aminopeptidase 1 (ERAP1), leading to reduced surface expression of MHC-I molecules 10. This impairment decreases CD8+ T cell recognition, allowing malignant cells to escape cytotoxic immune responses 11 (Table 3).

### Suppression of Pro-Inflammatory Cytokines

Wild-type P53 enhances anti-tumor immunity by regulating cytokine production. However, P53 mutations are associated with increased secretion of immunosuppressive cytokines such as IL-10 and TGF-β, which inhibit T cell activation and promote an immunosuppressive TME 12. Additionally, mutant P53 can upregulate PD-L1 expression, further dampening anti-tumor immune responses by suppressing T cell function 13 (Table 3).

### Recruitment of Immunosuppressive Cells

P53 mutations contribute to immune evasion by promoting the accumulation of myeloid-derived suppressor cells (MDSCs) and regulatory T cells (Tregs) within the TME 14. These cells inhibit cytotoxic T lymphocyte (CTL) function and facilitate tumor growth by secreting immunosuppressive factors and disrupting normal immune surveillance 15 (Table 3).

## P53 in Specific Hematologic Malignancies

### Acute Myeloid Leukemia (AML)

AML is a heterogeneous and aggressive malignancy characterized by clonal proliferation of myeloid precursors in the bone marrow 16. P53 mutations occur in approximately 5-10% of AML cases but are disproportionately represented in patients with complex karyotypes and therapy-related AML 17. These mutations are strongly associated with poor prognosis due to intrinsic resistance to conventional chemotherapy and a high risk of relapse 18. Mechanistically, mutant P53 contributes to immune evasion by downregulating antigen presentation machinery, increasing PD-L1 expression, and modulating cytokine secretion to create an immunosuppressive microenvironment 19 (Table 4).

### Myelodysplastic Syndromes (MDS)

MDS represents a group of hematologic disorders characterized by ineffective hematopoiesis and a high propensity for transformation into AML 20. TP53 mutations are found in approximately 7-11% of MDS cases and are associated with increased genomic instability and poor overall survival 21. The immunological consequences of P53 mutations in MDS include reduced MHC class I expression, leading to impaired antigen presentation, and increased secretion of immunosuppressive cytokines such as IL-6 and TGF-β. These factors contribute to a permissive bone marrow microenvironment that fosters malignant progression 22 (Table 4).

### Multiple Myeloma (MM)

Multiple myeloma (MM) is a malignancy of plasma cells characterized by clonal proliferation within the bone marrow, resulting in immune dysfunction and bone destruction 23. P53 mutations are present in 5-10% of newly diagnosed MM cases but become more frequent in relapsed and refractory disease, particularly in patients with chromosome 17p deletion (del(17p)) 24. In MM, IL-6 is excessively produced in the bone marrow, promoting the proliferation and survival of myeloma cells 25. Elevated IL-6 levels are associated with increased tumor burden, resistance to apoptosis, and poorer prognosis 26. Mutant P53 in MM may facilitate immune evasion by enhancing IL-6-driven tumor growth and suppressing T-cell responses. Furthermore, MM cells with impaired P53 function demonstrate heightened resistance to immune surveillance, underscoring the need for further research into potential counteracting strategies (Table 4).

### Lymphoma

P53 mutations are frequently observed in aggressive lymphomas, such as diffuse large B-cell lymphoma (DLBCL), mantle cell lymphoma (MCL), and Burkitt lymphoma, where they correlate with high tumor proliferation rates and immune escape 27. In DLBCL, TP53 mutations are associated with reduced immune surveillance due to impaired antigen presentation and increased expression of immune checkpoint molecules like PD-L1^28^. In MCL, TP53 mutations are a defining feature of blastoid and pleomorphic variants, which are highly aggressive and show increased immune evasion capabilities 29. Given the role of P53 in regulating immune responses, its dysfunction in lymphoma contributes significantly to tumor progression and resistance to immune-mediated tumor suppression.

### Chronic Lymphocytic Leukemia (CLL)

CLL is a malignancy of mature B lymphocytes and is one of the most common leukemias in adults 30. TP53 mutations occur in approximately 5-15% of newly diagnosed CLL cases but are significantly more prevalent in relapsed or refractory disease 31. These mutations often co-occur with 17p deletions, further impairing P53 function and leading to aggressive disease behavior and poor survival outcomes 31. P53 mutations in CLL contribute to immune evasion by altering antigen presentation, downregulating MHC class I expression, and increasing PD-L1 levels on leukemic cells 32. This results in impaired cytotoxic T cell activity and enhanced immune suppression within the tumor microenvironment 33. Additionally, mutant P53 influences cytokine secretion, promoting an immunosuppressive milieu through increased levels of TGF-β and IL-10, which inhibit T cell proliferation and function 34. Future research should focus on identifying biomarkers that predict disease progression and immune evasion mechanisms in CLL patients with TP53 mutations (Table 4).

## Therapeutic Strategies Targeting P53 and Immune Evasion

The tumor suppressor gene TP53, commonly referred to as the "guardian of the genome," plays a central role in maintaining cellular integrity by regulating the cell cycle, apoptosis, and genomic stability. Mutations or functional impairments in TP53 are present in more than 50% of human cancers and are often associated with poor prognosis, resistance to therapy, and increased immune evasion 35. Recent advancements have revealed novel strategies to reactivate or compensate for defective p53 signaling, with the dual goal of suppressing tumor proliferation and restoring anti-tumor immune responses. This section explores emerging therapeutic strategies focused on both wild-type and mutant forms of p53, with an emphasis on how these interventions modulate immune evasion in hematological malignancies such as acute myeloid leukemia (AML), myelodysplastic syndromes (MDS), chronic lymphocytic leukemia (CLL), and diffuse large B-cell lymphoma (DLBCL) (Table 5).

### Small-Molecule Activators

MDM2 Inhibitors (e.g., Idasanutlin), is one of the most promising classes of small-molecule activators targets MDM2, a negative regulator of p53. In normal cells, MDM2 binds to p53, promoting its ubiquitination and proteasomal degradation 36. In TP53 wild-type cancers, MDM2 is often overexpressed, leading to functional inactivation of p53 despite its intact genetic sequence 37. Idasanutlin, a potent and selective MDM2 antagonist, has shown efficacy in TP53 wild-type AML. By preventing MDM2-p53 interaction, Idasanutlin stabilizes p53, enhancing its transcriptional activity and promoting tumor cell apoptosis 38. Notably, Idasanutlin also restores MHC class I (MHC-I) expression on leukemia cells, which is essential for recognition by cytotoxic CD8+ T cells 39. This immune-modulating effect opens the door for synergistic combinations with immune checkpoint inhibitors or adoptive cell therapies. In the clinical trial NCT02545283, Idasanutlin achieved an overall response rate (ORR) of 40%, highlighting its potential in reactivating p53-dependent tumor suppressive and immune pathways 40.

APR-246 (Eprenetapopt), also known as Eprenetapopt, targets mutant p53 by restoring its proper folding and function 41. It is especially effective against common structural mutations such as R175H, which result in loss of DNA-binding capability. APR-246 promotes the refolding of mutant p53 into a functional conformation, allowing it to resume transcription of downstream targets involved in cell cycle arrest and apoptosis 42. Beyond its pro-apoptotic effects, APR-246 also modulates the immune landscape of tumors. It has been shown to downregulate PD-L1, a key immune checkpoint molecule that suppresses T cell activity 43. In a phase II clinical trial involving patients with MDS and TP53 mutations (Clinical trial NCT03745716), APR-246 demonstrated a complete remission (CR) rate of 30%, signifying its dual role in correcting p53 defects and mitigating immune escape mechanisms 44.

### Gene Therapy

CRISPR/Cas9-Mediated Gene Editing technologies such as CRISPR/Cas9 have opened new avenues for directly correcting TP53 mutations 45. In CLL models, CRISPR-based correction of TP53 mutations has led to restoration of wild-type p53 activity 46. This reactivation reinstates expression of miR-34a, a well-known p53-regulated microRNA that functions as a tumor suppressor and immune modulator. miR-34a targets several components of the PD-1/PD-L1 axis, and its expression correlates with reduced levels of PD-L1, thereby enhancing T cell-mediated anti-tumor responses 47. Though still preclinical, these findings suggest that gene therapy approaches could offer a curative potential by repairing the genetic defect at its source while simultaneously reawakening immune surveillance (Table 6).

### Adoptive Cell Transfer and Mutant Degraders

#### CAR-T Cell Therapy

Chimeric Antigen Receptor (CAR)-T cells represent a breakthrough in immunotherapy. However, patients with TP53 mutations often show resistance to conventional CAR-T approaches due to enhanced immune evasion and defective apoptosis 48. In DLBCL, tumors harboring TP53 mutations have been particularly refractory to CD19-targeted CAR-T cells 49. To overcome this, dual-targeting strategies such as CD19/CD22 CAR-T have been developed. These modified CAR-T cells recognize two different antigens simultaneously, reducing the risk of antigen escape 50. In the clinical trial NCT04007029, this dual-targeting approach resulted in a complete response rate of 58%, even in the presence of p53 dysfunction, suggesting that broadening antigen coverage can compensate for underlying genomic instability 51 (Table 7).

#### Arsenic Trioxide

Originally used in acute promyelocytic leukemia, arsenic trioxide has recently been investigated for its ability to degrade mutant p53 proteins in AML via the proteasomal pathway 52. This degradation eliminates the dominant-negative or gain-of-function effects of mutant p53, restoring the balance of cell cycle regulation and apoptosis 53. In trial NCT03855371, early data suggest that arsenic trioxide could selectively target mutant p53, potentially reversing immune evasion in AML by removing dysfunctional p53 isoforms that suppress immune gene expression 54.

GSK2830371, a selective inhibitor of PPM1D, has shown promise in preclinical AML models by restoring p53 function and increasing the transcription of pro-apoptotic genes 57-58. Interestingly, PPM1D inhibition also appears to reduce the expression of immune-inhibitory molecules, thereby sensitizing tumors to immune-mediated clearance 59. This strategy holds potential for AML patients with wild-type TP53 but dysregulated p53 signaling due to upstream suppressors like PPM1D (Table 8).

#### sEVs Delivering miR-34a

Small extracellular vesicles (sEVs) are emerging as efficient carriers for therapeutic nucleic acids 60. A novel strategy under investigation involves delivering miR-34a via liposomal formulations of sEVs. This approach takes advantage of miR-34a's ability to regulate both oncogenic pathways and immune checkpoint expression 61. In a Phase I trial (NCT05084365) involving patients with TP53-mutated CLL, systemically delivered miR-34a-containing sEVs have shown potential to reduce PD-L1 expression, suppress oncogenes, and promote immune activation 62. As this approach bypasses the need to correct the p53 gene directly, it represents an elegant method to re-establish downstream p53-like effects in tumors with irreversible TP53 mutations (Table 7).

Targeting TP53 and immune evasion mechanisms is a promising and rapidly evolving field, particularly for hematologic malignancies that have historically been difficult to treat due to p53 dysfunction. The strategies discussed—ranging from small-molecule reactivators and gene editing tools to advanced cell therapies and RNA-based interventions—highlight the diverse and complementary ways in which the p53 pathway can be modulated. A deeper understanding of p53's role in immune regulation is critical for designing effective combination therapies that harness both direct tumor suppression and immune activation, offering hope for improved outcomes in patients with p53-related malignancies.

## Discussion

The tumor suppressor p53 plays a pivotal role in maintaining genomic integrity, and its dysregulation is a hallmark of various hematologic malignancies, including AML, MDS, CLL, and DLBCL. While the clinical relevance of TP53 mutations has been extensively documented, our current study contributes to the field by highlighting emerging therapeutic strategies specifically designed to target p53 dysfunction and its downstream consequences on immune evasion.

One of the novel insights presented in this work is the therapeutic potential of MDM2 inhibitors, particularly Idasanutlin, in restoring MHC-I expression and enhancing immune surveillance in TP53 wild-type AML. This finding not only reinforces the immunomodulatory role of p53 but also supports the rationale for combining MDM2 inhibitors with immune checkpoint blockade therapies to augment antitumor immunity 36-40. Additionally, our discussion of APR-246 (Eprenetapopt) in correcting structural p53 mutations—especially in the context of PD-L1 downregulation in MDS—extends previous work by integrating its immunologic consequences, which had not been comprehensively addressed in earlier literature. By linking p53 restoration with checkpoint molecule suppression, we emphasize the dual impact of APR-246 on both tumor cell apoptosis and immune activation 41-44. Our study also underscores the therapeutic innovation of CRISPR/Cas9-mediated p53 repair, with a particular focus on its ability to re-induce miR-34a and suppress PD-L1 expression in CLL models. This mechanistic link between gene editing and immune checkpoint regulation provides a promising avenue for precision oncology in p53-mutant hematologic cancers 45-47. Moreover, we highlight dual-antigen targeting CAR-T strategies (CD19/CD22) as an effective approach to overcome immune resistance in TP53-mutated DLBCL. This represents a significant evolution beyond traditional CAR-T therapies, particularly for patients who fail standard treatments due to p53-related resistance pathways 48-51. Another novel aspect of our analysis includes the repurposing of arsenic trioxide for proteasomal degradation of mutant p53, expanding its utility beyond acute promyelocytic leukemia. This underexplored mechanism offers a valuable alternative for patients harboring dominant-negative or gain-of-function TP53 mutations 52-54. We also discuss the implications of targeting PPM1D overexpression, a common event in TP53 wild-type AML, with agents like GSK2830371. This sheds light on a non-mutational form of p53 suppression and illustrates how pharmacologic inhibition of p53 repressors may restore tumor suppressive function 55-59. Finally, we bring attention to a novel phase I trial of small extracellular vesicles (sEVs) delivering miR-34a, which represents a first-in-human effort to re-establish p53-related immune control via RNA-based nanomedicine in TP53-mutant CLL 60-62.

## Conclusion and Future Directions

P53 plays a pivotal role in hematologic malignancies by regulating immune surveillance and tumor suppression. Its mutations enable immune evasion, contributing to disease progression and therapy resistance. Understanding the mechanisms by which P53 mutations alter the immune landscape provides opportunities for developing targeted therapies. Future research should focus on refining combination strategies that integrate P53-targeted treatments with immunotherapy to improve patient outcomes in hematologic cancers.

## Figures and Tables

**Table 1:** Role of p53 in Normal Hematopoiesis and Immunity

Function	Description	Key Molecular Targets	Immune Relevance
Cell Cycle Arrest	Halts the cell cycle at G1/S checkpoint upon DNA damage	p21 (CDKN1A), GADD45	Prevents propagation of DNA-damaged HSCs
Apoptosis	Promotes mitochondrial-mediated apoptosis of damaged cells	BAX, PUMA, NOXA	Eliminates transformed hematopoietic cells
Senescence	Enforces terminal cell cycle arrest in pre-leukemic clones	p21, p16INK4A, pRB	Prevents clonal expansion of dysplastic cells
DNA Repair	Enhances nucleotide excision and base excision repair pathways	XPC, DDB2, p48	Maintains genomic stability
Immune Surveillance	Promotes antigen presentation and modulates cytokines (e.g., IFN-γ, IL-12)	TAP1/2, MHC-I, IRF9	Supports CD8+ T cell-mediated clearance

**Table 2:** Common TP53 Mutations in Hematologic Malignancies

Mutation (Codon)	Amino Acid Change	Structural/Functional Class	Associated Disease(s)	Frequency (%)	Effect on p53 Function
R175H	Arginine → Histidine	Conformational/structural	AML, MDS	~10-20	Loss of DNA binding, gain-of-function (GOF)
R248Q	Arginine → Glutamine	DNA contact mutation	CLL, DLBCL	~5-10	Disrupts target gene activation
R273H	Arginine → Histidine	DNA contact mutation	MDS, AML	~8-15	Retains protein stability, impairs transcription
G245S	Glycine → Serine	Structural destabilization	AML	~3-5	Destabilizes p53 conformation
Y220C	Tyrosine → Cysteine	Surface mutation	MDS	~2-4	Creates a destabilizing surface crevice
R282W	Arginine → Tryptophan	DNA contact mutation	DLBCL, MDS	~4-6	Inhibits proper folding

**Table 3:** Immune Evasion Mechanisms Associated with p53 Dysfunction

Mechanism	Effect of TP53 Loss or Mutation	Resulting Immune Impact
Downregulation of MHC-I	Reduces transcription of antigen-presentation machinery	Impaired CD8+ T cell recognition
Upregulation of PD-L1	Disinhibition of PD-L1 via miR-34a loss	T cell exhaustion and anergy
Impaired NK cell ligands	Reduced expression of NKG2D ligands	Decreased NK cell-mediated cytotoxicity
Altered cytokine secretion	Aberrant IL-6/IL-10 expression	Promotes suppressive myeloid-derived suppressor cells (MDSCs)
Loss of miR-34 family	Disruption of immune checkpoint control	Overexpression of PD-L1, CD47

**Table 4:** Hematologic Malignancies Frequently Involving TP53 Alterations

Malignancy	TP53 Mutation Frequency	Chromosomal Abnormalities	Prognosis When Mutated	Standard Therapy Response	Notable Features
AML	~10-20%	del(17p), complex karyotype	Very poor	Low CR rate (~20%)	Chemoresistance, early relapse
MDS	~5-15%	-5q, -7, del(17p)	Poor	Resistance to HMAs	Progresses to AML frequently
CLL	~8-15%	del(17p), del(11q)	Poor	Refractory to fludarabine	Prefer BTK/BCL2 inhibitors
DLBCL	~15%	17p deletions	Variable	Poor response to R-CHOP	Associated with double-hit lymphomas

**Table 5:** Small-Molecule Therapies Targeting p53 Pathway

Drug Name	Mechanism of Action	Cancer Type	Trial Phase	Clinical Trial ID	Response Rate	Additional Notes
Idasanutlin	MDM2 inhibitor (p53 stabilization)	AML (WT p53)	Phase II	NCT02545283	ORR: 40%	Synergistic with cytarabine
APR-246	Reactivates mutant p53 (R175H etc.)	MDS, AML	Phase III	NCT03745716	CR: 30%	Reduces PD-L1; combines with azacitidine
Arsenic Trioxide	Promotes degradation of mutant p53	AML	Phase I/II	NCT03855371	Ongoing	Synergy with proteasome inhibitors
Eprenetapopt	Re-folds mutant p53 to active form	MDS, AML	Phase II/III	Multiple	Varies	Induces ferroptosis

**Table 6:** Gene Therapy Approaches Targeting TP53

Strategy	Methodology	Disease Target	Outcome in Preclinical/Clinical Studies	Limitations
CRISPR/Cas9	Homology-directed repair of TP53	CLL, AML models	Restored p53 function, ↑miR-34a, ↓PD-L1	Delivery, off-target editing
AAV-p53 Delivery	Wild-type p53 via viral vector	MDS, AML	Induced apoptosis, tumor suppression	Immune clearance of vector
siRNA Knockdown	Silencing MDM2 or mutant TP53	CLL (in vitro)	Resensitization to chemotherapy	Transient effects

**Table 7:** Immunotherapy Strategies in p53-Mutant Hematologic Malignancies

Strategy	Target(s)	Disease	Clinical Trial/Study	Outcome	Considerations
CAR-T (CD19/CD22)	Dual-antigen CARs	DLBCL (TP53-mut)	NCT04007029	CR: 58%	Overcomes antigen escape
Checkpoint Blockade	PD-1/PD-L1, CTLA-4	AML, MDS	Multiple	Limited in TP53-mut	Often requires combination therapy
Liposomal miR-34a	miRNA replacement	CLL	NCT05084365	Phase I underway	Reduces PD-L1, targets oncogenes
BiTEs	CD3/CD19	B-ALL, CLL	Preclinical	Enhanced cytotoxicity	TP53 status may affect durability

**Table 8:** Emerging Small Molecules and Biological Agents in TP53 Pathway

Agent	Target	Disease Context	Mechanism	Development Status
GSK2830371	PPM1D (negative p53 regulator)	AML, MDS (WT p53 + PPM1D overexpression)	Restores p53-mediated apoptosis	Preclinical
COTI-2	Mutant p53, PI3K/mTOR	MDS, AML	Refolds mutant p53, inhibits mTOR	Phase I/II
Dual MDM2/MDMX Inhibitors	Nutlin-based derivatives	AML, CLL	Prevent full p53 degradation	Preclinical/early trials
